# Microporous Biocarbons Derived from *Inonotus obliquus* Mushroom and Their Application in the Removal of Liquid and Gaseous Impurities

**DOI:** 10.3390/ijms232415788

**Published:** 2022-12-13

**Authors:** Aleksandra Bazan-Wozniak, Judyta Cielecka-Piontek, Agnieszka Nosal-Wiercińska, Robert Pietrzak

**Affiliations:** 1 Faculty of Chemistry, Adam Mickiewicz University in Poznań, Uniwersytetu Poznańskiego 8, 61-614 Poznan, Poland; 2 Department of Pharmacognosy, Poznan University of Medical Sciences, Rokietnicka 3, 60-806 Poznan, Poland; 3 Faculty of Chemistry, Maria Curie-Sklodowska University in Lublin, Maria Curie-Sklodowska 3, 20-031 Lublin, Poland

**Keywords:** biocarbons, microwave and conventional heating, methyl red sodium salt, kinetic and equilibrium study, H_2_S adsorption

## Abstract

Biocarbons were obtained by physical and chemical activation of the residue of the extraction of chaga fungi (*Inonotus obliquus*). The residue was subjected to heat treatment carried out in a microwave oven and in a quartz tubular reactor. The materials were characterized by elemental analysis, low-temperature nitrogen adsorption, determination of pH, and the contents of acidic and basic oxygen functional groups on the surface of biocarbons by the Boehm method. The final biocarbon adsorbents have surface areas varying from 521–1004 m^2^/g. The physical activation of the precursor led to a strongly basic character of the surface. Chemical activation of *Inonotus obliquus* promoted the generation of acid functional groups. All biocarbons were used for methyl red sodium salt adsorption from the liquid phase. The sorption capacities of biocarbons towards the organic dye studied varied from 77 to 158 mg/g. The Langmuir model was found to better describe the experimental results. The results of the kinetic analysis showed that the adsorption of methyl red sodium salt on the biocarbons followed the pseudo-second-order model. The acidic environment was conducive to the adsorption of the dye on the obtained biocarbons. Moreover, thermodynamic studies confirmed that the organic dye adsorption on the biocarbons was a spontaneous endothermic process. The biocarbons obtained were also tested as adsorbents of hydrogen sulfide in dry and wet conditions. The sorption capacities towards hydrogen sulfide varied in the range of 21.9–77.9 mg. The results have shown that the adsorption of hydrogen sulfide depends on the process conditions and the activation procedure of biocarbons (method of activation and thermochemical treatment of samples). It has been shown that the initial material used can be a new precursor for obtaining cheap and—more importantly—universal bioadsorbents characterized by high effectiveness in the removal of air and water pollutants.

## 1. Introduction

Increasing environmental awareness has stimulated the search for adsorbents for the effective removal of gas [1,2,3,4,5,6,7] and liquid [8,9,10] pollutants and consequently, the interest in developing new technologies for the production of porous materials. According to many tests and laboratory experiments, adsorption on carbon adsorbents is one of the most effective methods of removal of liquid and gas pollutants from the natural environment [11,12]. Activated carbons are used as universal adsorbents thanks to their microporous structure and large surface area with a high degree of reactivity. They are non-toxic, can be regenerated and can be subjected to a variety of modifications, which determine their widespread use [13,14,15]. 

The microporous structure of carbon adsorbents permits the effective removal of gas pollutants; however, the prevalence of micropores in the adsorbent structure limits their performance as adsorbents of larger molecules of organic compounds. It should be emphasized that the effectiveness of pollutants removal depends not only on the specific surface area of the adsorbent but also on the presence of functional groups in the adsorbent’s structure, ash and a possible modification of the adsorbent’s surface [16,17,18,19,20,21]. 

The advantages offered by activated carbons as adsorbents are their low cost and rich gamut of resources. Industrial production of activated carbon employs two methods of activation: physical and chemical. The process of physical activation is based on carbonization, which is the thermal decomposition of the organic substance in a neutral gas atmosphere. The carbonizate is then subjected to activation in the presence of an oxidizing agent, usually carbon (IV) oxide. Chemical activation is based on the impregnation of a precursor with an activating agent and subjecting it to thermal treatment in a neutral gas atmosphere [22]. Effective adsorbents can be produced from biomass, which is beneficial as the products have a low content of ash, show high mechanical strength and are environmentally friendly. Production of carbon materials is continuously increasing, and new possibilities for their application are still emerging [23,24,25,26].

In this study, the precursor for the production of activated carbons were the residues of the extraction of chaga fungi (*Inonotus obliquus*). It is known that polysaccharides isolated from chaga fungi show a number of attractive properties such as anticancer, antioxidative, anti-inflammatory and increasing resistance [27]. The residue of the fungi extraction may be a new cheap precursor for the production of carbon adsorbents, first of all, activated carbons. An important feature of activated carbon adsorbents to be obtained is their universality, i.e., the effectiveness in the removal of pollutants from liquid and gas phases, as the majority of authors have focused on adsorption from one type of media. In our study, we also test the universality of the obtained adsorbents. 

**Figure 1:**
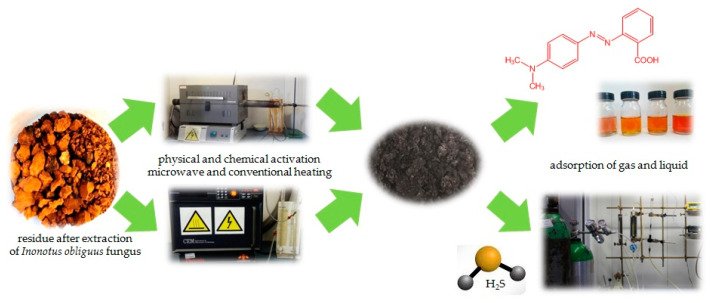
Scheme of the biocarbons preparation.

The main aim of the study was to obtain cheap and effective biocarbons from the residue of the extraction of chaga fungi (*Inonotus obliquus)* and its comprehensive characterization by the determination of textural parameters, the content of surface oxygen functional groups and pH of its water extract (Figure 1). The biocarbons were tested as potential adsorbents for the removal of liquid pollutants represented by methyl red sodium salt. The effectiveness of the dye adsorption on the surface of biocarbons was evaluated by determination of the effects of the initial concentration of dye solution, time of contact between the adsorbate and adsorbent, pH and temperature of the reaction system on the sorption capacities. Another series of experiments was performed to assess the effectiveness of the obtained biocarbons as adsorbents of gas pollutants, represented by hydrogen sulfide. The effects of the methods of activation and variant of heating (microwave and conventional) on the physicochemical and sorption properties of the biocarbons were also examined.

The paper is organized as follows. The Section 2 presents the elemental analysis of the obtained biocarbons, their textural parameters and the number of oxygen groups of acidic or basic character on the biocarbons’ surface. This section also presents the results of the adsorption of methyl red from water solution and gas hydrogen sulfide. In the section on Materials and Methods, we present the methods of biocarbon activation and heating along with the procedures used for the determination of the physicochemical and sorption properties of the biocarbons. The most important results and conclusions are given in the Section 4.

## 2. Results and Discussion

### 2.1. Characterization of the Biocarbons

Table 1 presents the results of the elementary analysis of the obtained biocarbons together with the efficiency of the activation processes. As follows from a comparison of results, the activation method and the mode of heating have little impact on the elemental composition of the biocarbons. The content of C^daf^ in the biocarbons varied in the range from 84.7 to 92.1 wt.%, the content of hydrogen from 1.8 to 2.7 wt.%, nitrogen from 2.9 to 4.4 wt.% and the content of sulfur did not exceed 0.4 wt.%. The content of O^daf^ varied from 3.1 to 8.0 wt.%.

As shown in Table 1, the content of mineral substances in the biocarbons obtained is low and varies from 5.4 to 1.9 wt.%. It is the lowest in samples ACm and ACc, which most probably follows from the fact that a large fraction of ash could have been removed as a result of the reaction between the precursor and activator (potassium carbonate). Moreover, the mineral substances could have been washed out from the carbon structure of the biocarbons in the process of their washing with a 5% solution of hydrochloric acid. Samples AFm and ACm obtained in a microwave oven contained more ash than the analogous samples produced by conventional heating (AFc and ACc). A low content of mineral substances is usually a desired feature in the application of biocarbons for adsorption from the liquid phase [28]. The efficiency of activation depends on the mode of sample heating and the type of activation method. 

The efficiency of the physical activation of the residue of the extraction of chaga fungi (*Inonotus obliquus*) varies in the range of 46.1–56.3% and is much higher than that of chemical activation. The fact of lower efficiency of chemical activation may be a consequence of the high reactivity of potassium carbonate toward the initial material. As to the effect of the mode of heating, the use of a microwave permits obtaining biocarbons with higher efficiency than the use of conventional heating.

Table 2 presents the structural and textural parameters characterizing the obtained biocarbons. According to these data, the surface area of the biocarbon samples depends on the method of activation and mode of heating.

The surface area of the biocarbons varies from 521 to 1004 m^2^/g. The largest surface area and pore volume were found for sample ACc, obtained by activation with potassium carbonate and subjected to conventional heating. The poorest textural parameters characterize sample AFm, whose surface area is a little greater than 500 m^2^/g, and the total pore volume is 0.53 cm^3^/g. According to Table 2 data, the physical activation leads to samples of poorer textural parameters than those of the samples subjected to chemical activation, i.e., ACm and ACc. Moreover, conventional heating was found to give samples a better-developed surface area, as follows from the higher values of textural parameters of samples AFc and ACc than those of AFm and ACm. All biocarbon samples obtained show microporous texture as the contribution of micropores is close to 90% of all pores in the samples. The highest contribution of micropores was observed for the chemically activated samples ACm and ACc (93%). The average pore diameters measured for all the samples varied from 2.34 to 3.42 nm.

**Figure 2:**
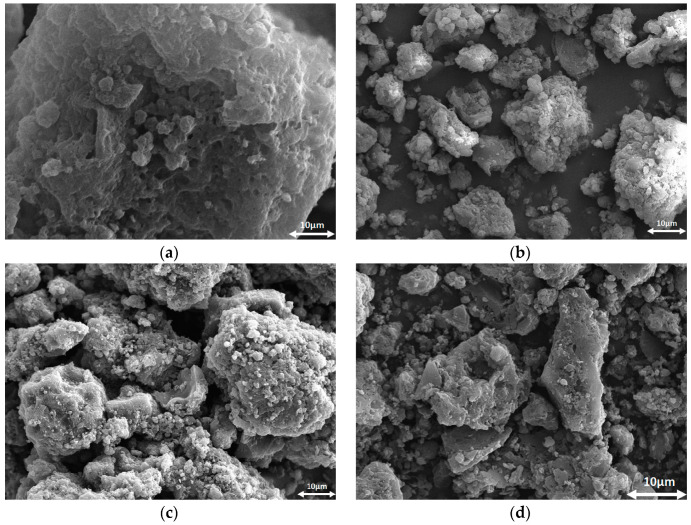
SEM micrographs of AFm (**a**), AFc (**b**), ACm (**c**) and ACc (**d**).

The SEM images of the obtained adsorbents are presented in Figure 2. The brighter fragments observed on the biocarbons may be due to the presence of ash.

The content of oxygen functional groups of acidic and basic character was measured by the Boehm titration method and the results are displayed in Table 3.

Their analysis reveals that the chemistry of the biocarbons’ surface depends first of all on the method of their production and to a much lower degree on the mode of heating. Samples AFm and AFc show definitely the basic character of their surfaces, which is confirmed by the values of the pH of their water extracts, 8.9 for AFm and 10.9 for AFc 10.9. The samples’ activation with potassium carbonate favors the generation of acidic functional groups. Determination of the content of oxygen functional groups by the Boehm method proved that sample ACc contained 1.52 mmol/g of acidic groups and 0.27 mmol/g of basic groups, while biocarbon ACm had the highest content of acidic groups of all samples, 2.11 mmol/g. To a certain degree, the chemical nature of the biocarbons’ surface also depended on the mode of heating, the use of microwave heating favoring the generation of a prevalence of acidic groups.

### 2.2. Adsorption of Methyl Red Sodium Salt

#### 2.2.1. Adsorption Isotherms of Methyl Red Sodium Salt

All biocarbons obtained were tested in the removal of methyl red sodium salt from water solutions. The results are presented in Table 4. The sorption capacities of the samples studied vary in the range from 77 to 158 mg/g. The efficiency of the sorption of the dye studied was observed to depend on the method of obtaining the biocarbons and the mode of their thermal treatment. The most effective adsorber was sample ACc, capable of adsorption of 158 mg of the dye, while the least effective was sample AFm. The sorption capacities of the biocarbons obtained by chemical activation were higher than those of the samples after physical activation. The amount of the dye adsorbed on biocarbons ACm and ACc varied in the range of 142–158 mg/g. The sorption capacity of AFm was lower by half or more than that of the chemically activated samples, and was 77 mg/g.

The sorption capacities of the biocarbons were also found to depend on the mode of heating and were higher for samples AFc and ACc (conventional heating) than for AFm and ACm (microwave heating). The impact of the mode of heating was higher on the samples obtained by a two-step physical activation of the precursor. For the microwave-heated biocarbons AFm and AFc, the sorption capacities were higher by 51 mg (AFm—77 mg/g, AFc—128 mg/g). The sorption capacities of the samples obtained by chemical activation of the residue of the extraction of chaga fungi and heated by microwave radiation and conventional heating differed only by 16 mg (ACm—142 mg/g, ACc—158 mg/g).

**Figure 3:**
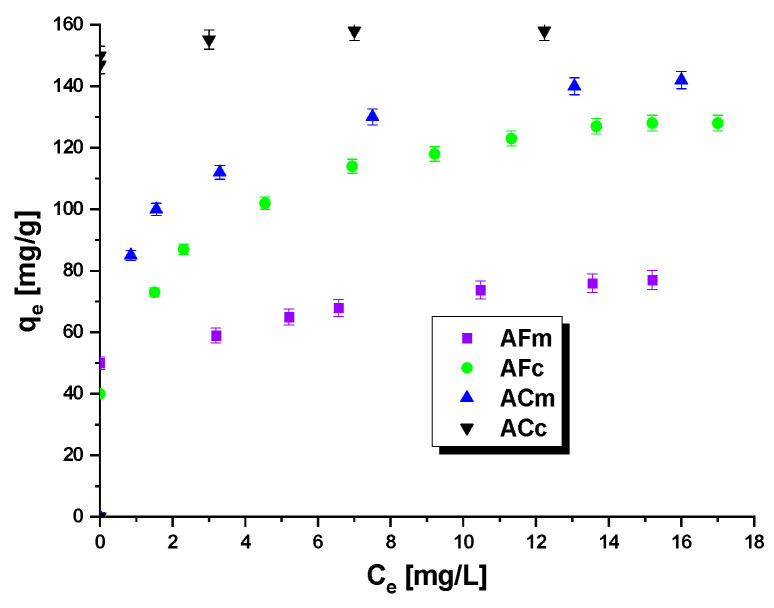
Adsorption of methyl red sodium salt onto biocarbons.

The isotherms of methyl red sodium salt adsorption obtained for all samples are shown in Figure 3. The amount of adsorbed dye depends on the initial concentration of the dye, i.e., it increases with increasing dye concentration. At low concentrations of methyl red sodium salt, the adsorption has a random character [29]; moreover, the number of active centers on the biocarbons’ surface is then much higher than that of the dye molecules, which is favorable for adsorption. When a certain dye concentration is exceeded, its molecules are closely packed on the biocarbon surface, which considerably hampers the guest–host interaction and leads to a state of adsorption equilibrium [30].

**Figure 4:**
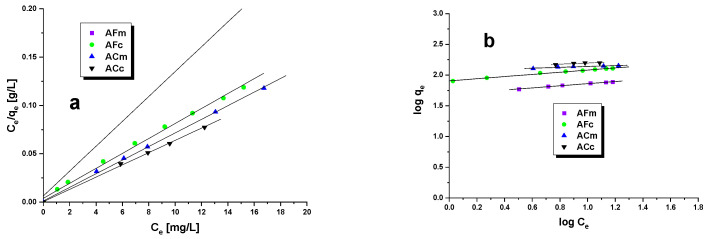
Langmuir (**a**) and Freundlich (**b**) isotherms onto biocarbons.

The methyl red sodium salt adsorption from its water solutions was interpreted assuming the linear forms of the Langmuir and Freundlich equations (Table 4, Figure 4a,b) [31]. The Langmuir model of adsorption is based on the assumption that on the adsorbent surface of the adsorbate a monomolecular layer is formed. It would mean that the surface of biocarbons has a certain number of active centers that are able to adsorb only one molecule of the adsorbate [32]. The Langmuir isotherm is represented by the following linear Equation (1):

(1) $$q_{e}=\frac{q_{m}\times K_{L}\times C_{e}}{1+(K_{L}\times C_{e})}$$

where *C_e_*—equilibrium dye concentration [mg/L], *q_e_*—equilibrium adsorption amount [mg/g], *K_L_*—Langmuir adsorption equilibrium constant [L/mg], and *q_max_*—maximum adsorption capacity of the adsorbent [mg/g].

The Freundlich model applies to heterogeneous systems and is based on the assumption that when the adsorbent surface is fully covered with the adsorbate molecules, the number of the adsorbed molecules cannot be greater than the number of available active centers on the adsorbent surface [33]. This model is described by the following Equation (2):

(2) $$q_{e}=K_{F}\times C_{e}{}^{1/n_{F}}$$

where *q_e_*—the amount of dye adsorbed at equilibrium [mg/g], *K_F_*—Freundlich equilibrium constant [mg/g × (mg/L)^1/n^], 1/n—the intensity of adsorption constant, and *C_e_*—equilibrium dye concentration [mg/L].

The choice of the model that more accurately describes the interactions between the adsorbate and adsorbent is made on the basis of the determination coefficient R^2^: the closer its value is to 1, the better the approximation with a given model. As concluded on the basis of Table 4 data, all the adsorption isotherms of methyl red sodium salt can be described by the Langmuir model. The determination coefficient for this model varies from 0.994 to 0.999. The R^2^ values obtained for the approximation with the Freundlich are lower and equal to 0.991 for AFm, 0.993 for AFc, 0.899 for ACm and 0.843 for ACc. The closer fit of the experimental data with the Langmuir curve suggests the formation of a monolayer of the adsorbate on the surface of the adsorbent. The Langmuir adsorption equilibrium constant K_L_ is related to the adsorption capacity of the adsorbent, and the higher its value, the greater the selectivity of the adsorbent to the adsorbate. The highest K_L_ value (0.079) was obtained for the sample produced by chemical activation under conventional heating ACc, which means that this biocarbon is the most selective to the dye studied. Analysis of Table 4 data also shows that the maximum sorption capacities calculated from the Langmuir equation q_max_ are close to those obtained from the experimental results. In the Freundlich equation, the constant 1/n is the inhomogeneity factor depending on the affinity of the adsorbate to the adsorbent. The constant 1/n can be determined from the Freundlich isotherm; the closer it is to zero, the greater the affinity. On the basis of Table 4 data, it can be concluded that the greatest affinity to the adsorbate is shown by the samples obtained as a result of the chemical activation of the precursor.

Table 5 presents the values of the sorption capacities obtained for the biocarbons studied and the literature data for other adsorbents. According to Table 5 data, the biocarbons obtained from the residue of the extraction of chaga fungi (*Inonotus obliquus*) are superior relative to the parameters obtained for NaAlg-Chit/nZVI [34] and thiosemicarbazide modified chitosan [31]. The adsorbent NaAlg-Chit/nZVI [34] was obtained from iron nanoparticles and sodium alginate chitosan. Methyl red adsorption on this nanocomposite [34] has shown that this process occurred according to the Langmuir model, and the value of q_max_ for this adsorbent was 9.48 mg/g. Moreover, with regard to thiosemicarbazide modified chitosan, whose maximum sorption capacity toward methyl red was 17.31 mg/g, the adsorption occurred according to the Langmuir model [35]. A much greater sorption capacity has been reported for biogenic Ag@Fe nanocomposite [36], although it was lower than those of AFm, ACm and ACc obtained from the residue of the extraction of chaga fungi. According to Table 5, a much greater sorption capacity towards methyl (over twice greater than that of Acc) has been achieved by mesoporous activated carbon from durian seed [37]. The biocarbon adsorbent was obtained as a result of the chemical activation of durian seeds by KOH. The adsorption on this adsorbent was best fitted with the Freundlich model, so it had a multilayer character. 

#### 2.2.2. Effect of Agitation Time on the Adsorption

**Figure 5:**
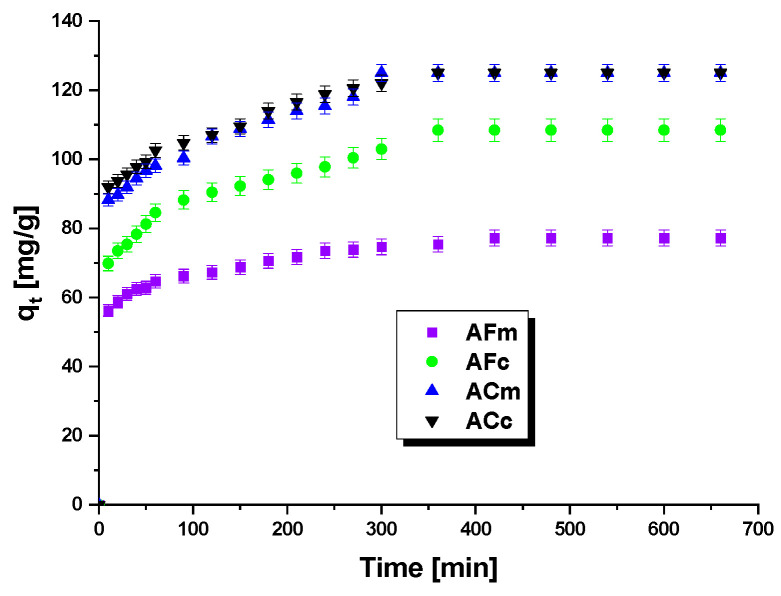
Influence of contact time on adsorption of methyl red sodium salt on biocarbons.

The effect of the time of contact of the dye solution with the biocarbon on the effectiveness of adsorption was also characterized. The results are presented in Figure 5 and imply that the effect of the time of contact between the adsorbent and the adsorber has a significant influence on the sorption capacity of the biocarbons studied. In the first 30–40 min of stirring the methyl red sodium salt was adsorbed very quickly on the biocarbons, most probably because of a large number of free active centers on their surfaces. With increasing time of contact, the number of free active centers decreases, and their gradual saturation is observed. Moreover, the active centers become less accessible because of the repulsive interactions between the adsorbed dye molecules and those that are in the solution [39]. As follows from the course of the isotherms, the state of adsorption equilibrium is reached after 300–350 min, which means that at that time there are no free active centers, and the sorption capacity does not increase.

The kinetics of methyl red sodium salt adsorption on the surface of the biocarbons studied was approximated by two models: pseudo-first-order and pseudo-second-order. The pseudo-first-order model [40] can be expressed by the following Formula (3):

(3) $$logq_{e}=logK_{F}+\frac{1}{n}logC_{e}$$

where *q_e_*, *q_t_*—amounts of dye adsorbed [mg/g], *t*—time [min], and *k*_1_—pseudo-first order adsorption constant [1/min].

The pseudo-second-order model [41] can be expressed by the following linear form (4):

(4) $$\frac{t}{q_{t}}=\frac{1}{k_{2}q_{e}{}^{2}}+\frac{t}{q_{e}}$$

where *k*_2_—second order reaction rate equilibrium constant [g/mg×min].

**Figure 6:**
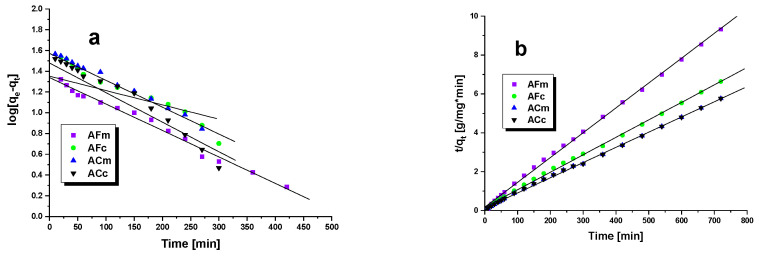
Pseudo-first-order (**a**) and pseudo-second-order (**b**) kinetic plots for adsorption of methyl red sodium salt onto biocarbons.

The kinetic parameters of these models are given in Table 6 and the curves obtained on the basis of these data are presented in Figure 6a,b.

As follows from the Table 6 data, the coefficient of determination R^2^ for the pseudo-first-order kinetics model varied from 0.973 to 0.995, while its value for the pseudo-second-order model was 0.999 for each sample. Thus, the adsorption of the dye on the biocarbons surfaces occurs according to the pseudo-second-order model. Moreover, the sorption capacities (q_e_) are close to the values of q_e(cal)_ calculated from the pseudo-second-order equation, while for the pseudo-first-order model these values are significantly different.

#### 2.2.3. Effect of pH and Temperature on the Adsorption

**Figure 7:**
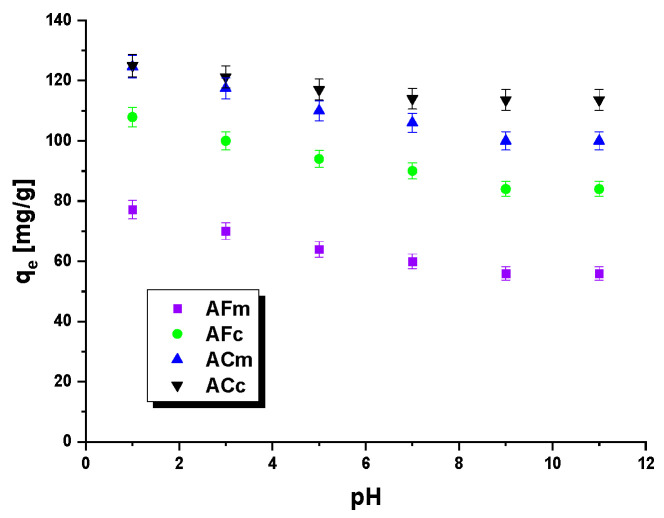
Effect of pH on the removal of methyl red sodium salt by biocarbons.

The next task was to check the effect of the pH of the dye solution on the sorption capacities of the biocarbons obtained. The results measured for the dye solvent pH values from 1 to 11 are displayed in Figure 7. The plots of sorption capacities as a function of pH values (Figure 7) show that at pH 1 the capacities were the highest. With increasing pH, the effectiveness of the removal of methyl red sodium salt decreased. The most pronounced decrease, by 24 mg/g, was noted for the sample obtained as a result of chemical activation and heated by microwave irradiation, ACm. In solutions with low pH, the dominant ions were H^+^, which enhance the positive charge on the adsorbent surface, promoting the adsorption of the anionic dye. According to the literature data, at low pH, the removal efficiency of adsorbed dye is higher, while at high pH it is lower [42].

**Figure 8:**
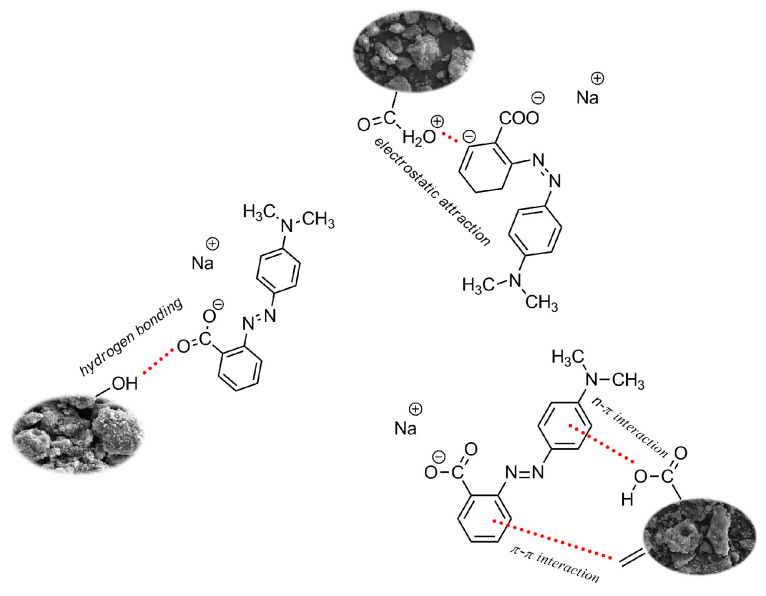
Organic dye adsorption on biocarbons.

The pH_pzc_ values of the biocarbons studied were determined by the drift method in the pH range 2–12, to be 6.4 for AFm, 6.8 for AFc, 4.1 for ACm and 5.6 for ACc. As mentioned above, the biocarbon surfaces host acidic and basic groups, so electrostatic interactions and the formation of hydrogen bonds may occur during adsorption. The mechanism of adsorption of the dye may also involve the π–π and n–π interactions (Figure 8).

**Figure 9:**
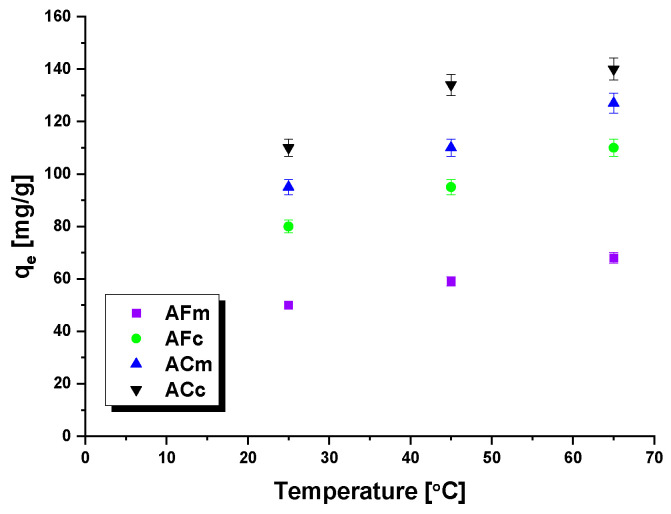
Effect of temperature on the adsorption of methyl red sodium salt onto biocarbons.

The impact of temperature on the sorption capacity of the biocarbons studied towards methyl red sodium salt was also evaluated and the results are given in Figure 9. The efficiency of the dye removal was shown to increase with increasing temperature, which is related to the greater mobility of methyl red sodium salt in solution at higher temperatures [43]. The most pronounced effect of temperature on the biocarbon sorption capacity was observed for sample ACm. With the temperature increasing from 25 to 65 °C, its sorption capacity increased by over 30 mg. The smallest temperature impact was noted for sample AFm (physical activation, microwave irradiation), whose capacity increased only by 17 mg upon the same increase in temperature.

In order to describe the energy effects related to adsorption, a number of thermodynamical parameters were calculated, including standard Gibbs enthalpy (Δ*G*^0^), entropy (Δ*S*^0^) and standard enthalpy of adsorption (Δ*H*^0^), and the results are shown in Table 7.

The values of Δ*S*^0^ and Δ*H*^0^ were calculated from the Formula (5):

(5) $$lnK_{d}=\frac{\Delta S^{0}}{R}-\frac{\Delta H^{0}}{RT}$$

where *T*—absolute temperature [K], *R*—universal gas constant (8.314 [J/K × mol]), while *K_d_* is defined as (6):

(6) $$K_{d}=\frac{q_{e}}{C_{e}}$$

The values of Δ*S*^0^ and Δ*H*^0^ were read off the plot of ln *K_d_* versus 1/T. The value of Δ*G*^0^ was calculated as (7):

(7) ΔG=ΔH−TΔS

The negative values of free enthalpy mean that the process of sorption of the dye is spontaneous in character. When the temperature of the process increases, the value of ΔG^0^ decreases. Positive values of the standard enthalpy of adsorption imply that the adsorption of methyl red sodium salt is an endothermic process [44]. It can be also inferred that the increase in temperature enhances the degree of spontaneity of the reaction.

### 2.3. Adsorption of H_2_S

**Figure 10:**
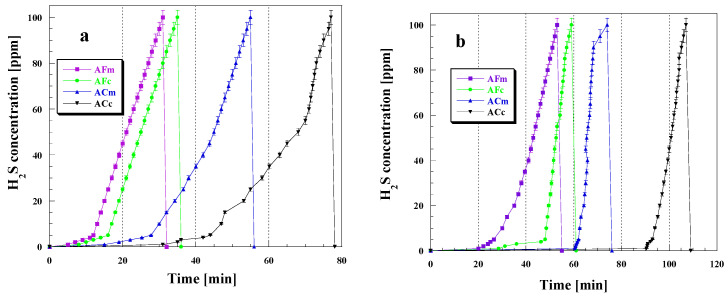
H_2_S breakthrough curves for the biocarbons studied in dry (**a**) and wet (**b**) conditions.

The obtained biocarbons were also tested in the removal of a gas pollutant, hydrogen sulfide (Table 8, Figure 10).

The effectiveness of hydrogen sulfide removal was found to depend on the conditions of the process, method of sample activation and mode of its heating. For all biocarbon samples studied, higher sorption capacities were obtained in wet conditions, which is a consequence of the formation of a water film on their surfaces [45]. The greatest difference between sorption capacity values measured in wet and dry conditions was detected for sample ACm (chemical activation, microwave irradiation). When the adsorption was carried out in wet conditions, for all biomaterials the time of breakthrough of the carbon bed was longer (Figure 10b). The greatest difference between the time of breakthrough in wet and dry conditions was observed for sample ACc for which it was 31 min in dry conditions and 90 min in the presence of steam. The same sample proved to be the most effective in the adsorption of hydrogen sulfide, irrespective of the conditions of the process. The sorption capacity of this sample in dry conditions was 55.3 mg, while in wet conditions it was 77.8 mg. The efficiency of hydrogen sulfide removal also depended on the surface area and degree of porous structure development of the biocarbons; the larger the surface area and the more developed the porous structure, the greater the sorption capacity toward H_2_S (see the data in Table 2 and Table 8). According to the literature data, the mechanism of H_2_S adsorption on the surface of the biocarbons depends on pH [45]. A too-low pH of the sample hinders the hydrogen sulfide dissociation, while a too-high value leads to the presence of polysulfides and elemental sulfur. When the pH is moderate (slightly acidic), sulfur may be oxidized to sulfur dioxide [45]. The biocarbons obtained by chemical activation of the residue of the extraction of chaga fungi (*Inonotus obliquus*) have an acidic surface so they showed the highest sorption capacities. However, it should be noted that the chemical character of the sample is not the only parameter determining hydrogen sulfide adsorption as it also depends on the conditions of the process and degree of the surface area development.

Analysis of the time changes in hydrogen sulfide concentration reveals that after the breakthrough of the carbon bed, for all biocarbon samples studied the concentration of H_2_S increases relatively quickly to the maximum value of 100 ppm. This process was the longest in dry conditions for the sample subjected to chemical activation. The shapes of the time dependences of hydrogen sulfide concentration are very similar and irrelevant of the conditions of the process, which indicates that the presence of steam in the gas flow has no significant effect on the mechanism of H_2_S sorption. After cutting off the H_2_S inflow to the carbon bed, for all samples, a significant decrease in the concentration of this pollutant was observed in 5 min. This means that the majority of the adsorbed hydrogen sulfide has been strongly bound in the porous structure or has been chemisorbed [45]. The sorption abilities of the biocarbons studied also depend on the method of their activation as higher adsorption capacities were obtained for the samples obtained by chemical activation of the precursor. The effect of the activation method is particularly pronounced for the samples heated in a conventional furnace when the H_2_S adsorption is carried out in dry conditions. The sorption capacity of sample ACc was over twice greater than that of AFc. As mentioned earlier, another factor affecting the sorption capacities is the mode of the sample heating. Irrespective of the adsorption conditions, the sorption capacities of the samples heated by the conventional method were higher than those of the samples subjected to microwave irradiation. The differences were greater when adsorption takes place in wet conditions.

The biocarbons obtained in this study show higher sorption capacities towards H_2_S than the other biomaterials investigated earlier by our group (Table 9) [46]. The biocarbons obtained by physical activation of the residues of supercritical extraction of marigold flowers and hop cones [46] were able to adsorb 29.6 and 17.1 mg H_2_S, respectively. The measurements were made in the mix wet conditions, so at first, the samples were wetted for 30 min in a stream of air of 70% humidity and then adsorption of H_2_S was carried out in wet conditions. Much higher effectiveness in H_2_S removal showed the biocarbon obtained from commercial coconut shell [47], whose sorption capacity was almost 30 mg greater (109.3 mg/g) than that of our Acc sample (77.8 mg/g) produced by chemical activation of the residue of the extraction of chaga fungi and subjected to conventional heating. Wang et al. [48] have studied H_2_S adsorption on the activated carbon obtained by chemical activation with KOH of the residue of coffee extraction. The surface area of this adsorbent was enriched by impregnation with copper and its sorption capacity was 132.22 mg/g. Wang et al. [48] have shown the positive impact of the functional groups generated as a result of impregnation with copper on the rate of H_2_S adsorption.

## 3. Materials and Methods

### 3.1. Precursors and Activated Biocarbons Preparation

The precursor of the biocarbons studied was the residue of the extraction of chaga fungi (*Inonotus obliquus*). The chemical activation process was carried out using analytical purity potassium carbonate which was purchased from Stanlab (Poland). Methyl red sodium salt was used to prepare the 1000 mg/L stock solution of organic dye used in all adsorption experiments. All chemicals were of analytical reagent grade and were used directly without any further purification.

The starting material in a form of powder with a grain size range of 0.80–1.75 mm and moisture content in the air-dry range of 8.5 wt.% was subjected to physical or chemical activation. The heat treatment was carried out in a microwave oven (m) or in a quartz tubular reactor (c).

The process of pyrolysis was carried under a stream of nitrogen. The starting material was heated at the rate of 5 °C/min from room temperature to the final temperature of 500 °C, kept for 60 min at that temperature and then cooled in an inert atmosphere to room temperature. The physical activation of pyrolysis products was carried at 700 °C under a stream of carbon(IV) oxide for 30 min in a microwave oven (AFm) or for 60 min in a quartz tubular reactor (AFc).

The precursor also was impregnated with K_2_CO_3_ solution (weight ratio of precursor to activator of 1:2), dried to constant mass at 110 °C and then subjected to chemical activation in a nitrogen atmosphere. The impregnated samples were heated (5 °C/min) from room temperature to 800 °C, kept at the final activation temperature for 30 min (microwave oven, ACm) or 45 min (conventional oven, ACc) and then cooled down in nitrogen flow room temperature. The final products were subjected to two-step washing procedure, firstly with a hot 5% solution of HCl and later with demineralized water until free of chloride ions. Finally, the samples were dried to constant mass at 110 °C.

### 3.2. Sample Characterization

#### 3.2.1. Elemental Analysis

The elemental analysis of the biocarbons was performed by using the Vario EL III elemental analyzer (Elementar Analysensysteme GmbH, Langenselbold, Germany). The standard test for ash was performed according to the ASTM D2866-94 Standard (2004). Elemental analyses of each of the obtained biocarbons were repeated twice.

#### 3.2.2. Nitrogen Sorption

The porosity was measured by using the nitrogen adsorption/desorption method. The measurement was made using an AutosorbiQ analyzer made by Quantachrome (Boynton Beach, FL, USA). The surface area of biocarbons was found on the basis of the multilayer adsorption BET (Brunauer-Emmett-Teller) theory. The pore size distribution and total pore volume were determined on the basis of the BJH (Barrett-Joyner-Halenda) model. The micropore volume and surface area were found by the t-plot method. The specific surface area of each sample is the average of two measurements.

The SEM images of adsorbents were obtained using a scanning electron microscope Quanta 3D FEG (FEI, Field Electron and Ion Co., Oregon, USA).

#### 3.2.3. Acid–Base Properties

The pH of biocarbons was measured using the following procedure: 0.4 g of each sample was added to 20 mL of distilled water and the suspension was stirred overnight to reach equilibrium. After that, pH of the suspension was measured. The content of surface oxygen functional groups was determined according to the Boehm method [26]. For each biocarbon obtained a series of three measurements was made.

### 3.3. Adsorption Studies

To determine the adsorption capacity of the biocarbons adsorption, tests were conducted using model aqueous solutions of methyl red sodium salt. A stock solution of 1000 mg/L of the adsorbate was prepared and thereafter, working solutions were made to the desired concentrations through serial dilution. The influence of the experimental parameters, such as the initial concentration, pH and adsorption temperature on the sorption capacity of the biocarbons obtained was investigated in detail. All experiments were performed at 25 °C in triplicate. The amount of the adsorbate that was absorbed at equilibrium was calculated using the equation:

(8) $$q_{e}=\frac{C_{0}-C_{e}}{m}\times V$$

where: *C*_0_—initial methyl red sodium salt concentration [mg/L], *C_e_*—equilibrium methyl red sodium salt concentration [mg/L]; *m*—weight of the biocarbon [g], *V*—volume of the solution [L].

The evaluation of H_2_S sorption capacity: samples in the form of granules (0.80–1.75 mm in diameter) were packed into a glass column (length 300 mm internal diameter, 9 mm bed volume 3 cm^3^). The samples were tested in dry and wet conditions. Dry or wet air (70% humidity) with 0.1% of H_2_S was passed through the bed of the adsorbent at 0.450 L/ min. The concentration of H_2_S was monitored using a Multi-Gas Monitor. The tests were stopped at the breakthrough concentration of 100 ppm (for H_2_S) because of the electrochemical sensor limits. The capacities of each sorbent in terms of milligrams of H_2_S per gram of adsorbent were calculated according to the procedure [6]. The results are the averages of three measurements.

## 4. Conclusions

The above-presented results permit drawing a conclusion that the biocarbons obtained by physical and chemical activation of residues of *Inonotus obliquus* extraction with the use of conventional and microwave heating, can be effective adsorbents of liquid and gas pollutants. A great advantage of the biocarbons obtained from the residue of chaga fungi extract is their universality as the majority of adsorbents reported in the literature and used in industry are applied for the removal of pollutants from a single (gas or liquid) phase.

The surface area and degree of porous structure development were found to depend on the method of activation and mode of heating. The largest specific surface area was determined for the biocarbon obtained by chemical activation of the precursor under conventional heating (1004 m^2^/g). The conventional heating method and chemical activation were shown to provide adsorbents with a better-developed specific surface area. The chemical character of the surfaces of the biocarbons produced was found to depend, first of all, on the method of the sample activation: physical activation favors the formation of basic groups, while chemical activation leads to the prevalent formation of acidic groups.

The most effective adsorbent from among the samples studied was that obtained by chemical activation and conventional heating (158 mg/g), while the least effective one was that obtained by physical activation and heated in a microwave oven (77 mg/g). The effectiveness of methyl red sodium salt removal from its water solutions depended on the method of activation, mode of heating, initial concentration of the dye, time of contact of the adsorbent and the adsorber, pH and temperature of the reaction. The biocarbons obtained by chemical activation of the precursor were more effective in the removal of methyl red than those obtained by physical activation. The biomaterials subjected to conventional heating showed greater sorption capacities than the samples obtained with the use of microwave heating. The adsorption equilibrium was achieved in about 300–350 min. All isotherms of the dye adsorption were found to be of the Langmuir type (R^2^ 0.994–0.999). Acidic environment is favorable for the dye adsorption. With increasing temperature, the activation barrier is overcome and the sorption capacity of the obtained biocarbons increases. The negative values of standard Gibbs enthalpy (ΔG^0^) and the positive values of standard enthalpy of adsorption (ΔH^0^) mean that the process of dye adsorption is spontaneous and endothermic. The sorption capacities of the studied biocarbons towards hydrogen sulfide depend on the conditions of the process, method of activation and mode of heating.

The sorption capacities towards H_2_S are in the range of 21.9–77.8 mg/g. The most effective adsorbent of H_2_S was the sample obtained as a result of chemical activation of the precursor subjected to conventional heating, which was also the most effective adsorbent of methyl red. The sorption capacities were greater when adsorption took place in the presence of steam. The other factors conducive to high sorption capacities are chemical activation and heating by the conventional method.

## Figures and Tables

**Table 1:** Elemental analysis of the biocarbons obtained and the yield of activation processes [wt.%].

Sample	C^daf 1^	H^daf^	N^daf^	S^daf^	O^daf^ *	Ash	Yield
AFm	84.7	2.7	4.4	0.2	8.0	5.4	56.3
AFc	88.6	2.1	3.9	0.4	5.0	4.5	46.1
ACm	89.5	2.0	3.8	0.2	4.5	2.1	28.9
ACc	92.1	1.8	2.9	0.1	3.1	1.9	21.9

^daf^—dry-ash-free basis; ^1^ method error ≤ 0.3%; *—determined by difference.

**Table 2:** Textural parameters of the biocarbons obtained.

Sample	Surface Area ^1^ [m^2^/g]	Micropore Area [m^2^/g]	Total Pore Volume [cm^3^/g]	Micropore Volume [cm^3^/g]	Average Pore Diameter [nm]
AFm	521	443	0.53	0.46	3.42
AFc	732	699	0.55	0.49	3.23
ACm	901	802	0.58	0.54	2.34
ACc	1004	981	0.61	0.57	2.96

^1^ error range between 2–5%.

**Table 3:** Acid-base properties of the biocarbons obtained.

Sample	Acidic Groups [mmol/g]	Basic Groups [mmol/g]	pH
AFm	0.22 ± 0.01	3.15 ± 0.03	8.9 ± 0.2
AFc	0.12 ± 0.01	4.29 ± 0.04	10.9 ± 0.3
ACm	2.11 ± 0.02	0.27 ± 0.01	3.5 ± 0.01
ACc	1.52 ± 0.02	0.33 ± 0.01	4.2 ± 0.01

**Table 4:** Langmuir and Freundlich parameters of the adsorption isotherms of methyl red sodium salt onto biocarbons.

Sample	q_e_ [mg/g]	Langmuir			Freundlich		
		R^2^	q_max_ [mg/g]	K_L_ [L/mg]	R^2^	K_F_ [mg/g(L/mg)^1/n^]	1/n
AFm	77	0.994	78	0.026	0.991	48.75	0.171
AFc	128	0.996	130	0.015	0.993	80.35	0.175
ACm	142	0.999	143	0.041	0.899	117.22	0.070
ACc	158	0.999	159	0.079	0.843	126.47	0.093

**Table 5:** Comparison of sorption capacities (methyl red) of biocarbons with other adsorbents presented in the literature.

Adsorbent	Sorption Capacity [mg/g]	Reference
biocarbons	77–158	This study
NaAlg-Chit/nZVI	9.48	[34]
thiosemicarbazide modified chitosan	17.31	[35]
biogenic Ag@Fe nanocomposite	125	[36]
mesoporous activated carbon from durian seed	384.62	[37]
lemongrass	76.923	[38]

**Table 6:** Adsorption kinetics parameters for the adsorption of methyl red sodium salt on the biocarbons studied.

Sample	q_e_ [mg/g]	Pseudo-First-Order Model			Pseudo-Second-Order Model		
		R^2^	k_1_ [1/min]	q_e,cal_ [mg/g]	R^2^	k_2_ [g/mg×min]	q_e,cal_ [mg/g]
AFm	77	0.988	5.76 × 10^−3^	22	0.999	9.63 × 10^−4^	78
AFc	108	0.973	5.99 × 10^−3^	37	0.999	4.15 × 10^−4^	112
ACm	125	0.995	6.22 × 10^−3^	40	0.999	4.96 × 10^−4^	128
ACc	125	0.974	7.83 × 10^−3^	39	0.999	5.87 × 10^−4^	128

**Table 7:** Thermodynamic parameters characterizing the adsorption of methyl red sodium salt on the biocarbons studied.

Sample	Temperature [°C]	∆*G*^0^ [kJ/mol]	∆*H*^0^ [kJ/mol]	∆*S*^0^ [J/mol×K]
AFm	25	−3.99	17.09	70.61
	45	−5.28		
	65	−6.82		
AFc	25	−5.41	18.91	81.50
	25	−5.41		
	45	−6.90		
ACm	25	−6.46	23.70	100.93
	45	−8.17		
	65	−10.53		
ACc	25	−7.20	32.06	123.05
	45	−9.28		
	65	−11.93		

**Table 8:** H_2_S breakthrough capacities of the biocarbons obtained.

Sample	Dry Conditions [mg/g]	Wet Conditions [mg/g]
AFm	21.9 ± 2.3	37.9 ± 2.7
AFc	24.8 ± 2.4	42.7 ± 8.8
ACm	30.3 ± 2.5	53.5 ± 9.1
ACc	55.3 ± 9.0	77.8 ± 9.4

**Table 9:** Comparison of sorption capacities (H_2_S) of biocarbons with other adsorbents reported in literature.

Adsorbent	Sorption Capacity [mg/g]	Reference
biocarbons	21.9–77.8	This study
biocarbon from marigold	29.6	[46]
biocarbon from hops	17.1	[46]
commercial coconut shell activated carbon	109.3	[47]
activated carbon from coffee residue	132.22	[48]
fly ash	15.89	[49]

## Data Availability

Data are contained within the article.
